# Beyond Darwin: evolvability and the generation of novelty

**DOI:** 10.1186/1741-7007-11-110

**Published:** 2013-11-07

**Authors:** Marc Kirschner

**Affiliations:** 1 Department of Systems Biology, Harvard Medical School, Warren Alpert Building, 200 Longwood Avenue, Boston, MA 02115, USA

## 

Marc Kirschner graduated in biochemistry from Northwestern University, moving to Berkeley for his doctoral research and with positions at Berkeley, Oxford University and Princeton before he took a professorship at University of California San Francisco where with Andrew Murray he did seminal research on the control of the cell cycle in *Xenopus* egg extracts that led to the discovery of how cyclin drives the cell cycle, and with Tim Mitchison on the dynamic instability of microtubules. In 1993 he moved to Harvard where in 2003 he became the founding Chair of the HMS Department of Systems Biology and was named the John Franklin Enders University Professor in 2009. The two books he wrote with John Gerhart, *Cells, Embryos and Evolution* (Blackwell, 1997) and *The Plausibility of Life: Resolving Darwin’s Dilemma* (Yale University Press, 2005), reflect his deep and longstanding interest in how biological systems evolve. Here he gives his view of the evolution of evolvability and its profound importance for understanding and applying biology.

**Figure:**
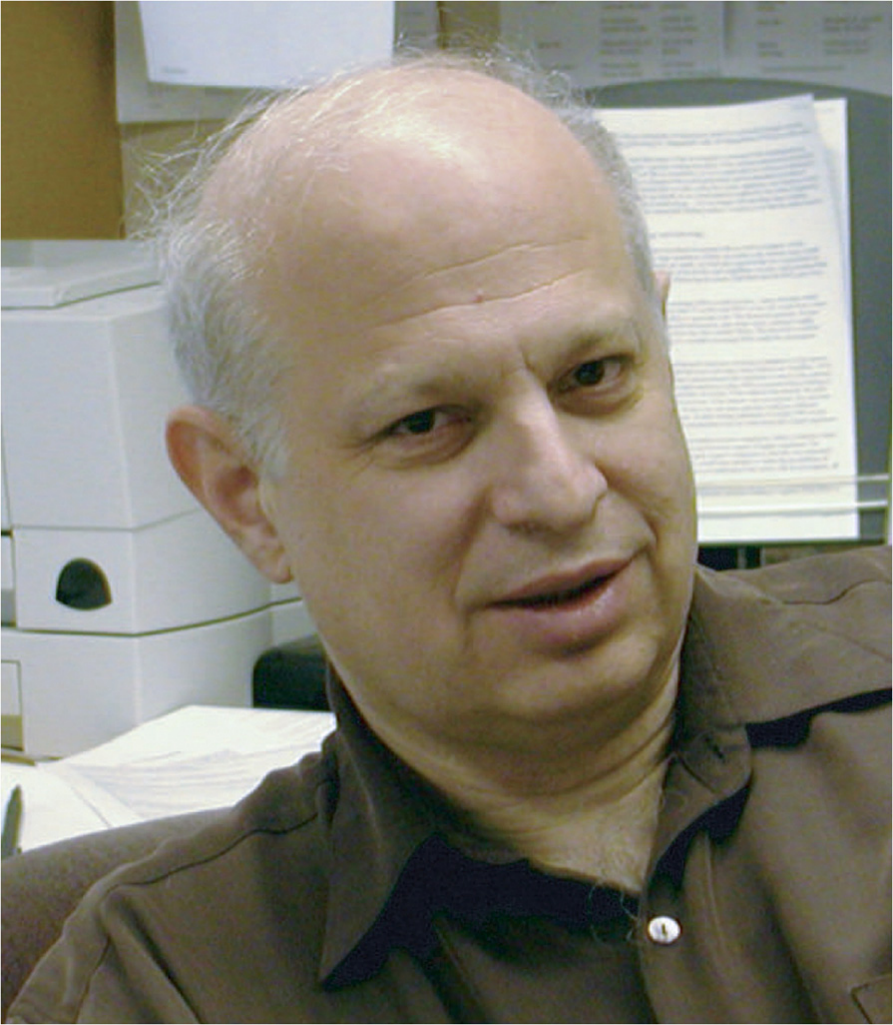
Marc Kirschner

## What is evolvability - how would you define it?

In some sort of tautological way evolvability is simply the capacity of a system to evolve. But more than that, in a Darwinian sense it speaks to both the amount of variation that is subject to selection, and the nature of that variation. And if there is something like evolvability, that would mean that systems are constructed in such a way that they generate a lot of phenotypic variation on which selection can act with a given amount, or a minimum amount, of genetic variation. But the variation must be variation of a special type: first of all, it has to be non-lethal because if it’s lethal it isn’t contributed to the next generation; and second, I would argue that it is a kind of variation that is more likely to be functional even for circumstances never previously encountered by the organism. That’s a little more difficult to explain, but effectively what I mean is that if you make random changes in mechanical systems they inevitably either have no effect or they break the system, whereas biological systems are able to survive random changes sometimes better, or often not so much worse; so biological systems are unusual in that you can make random changes and the system still functions, allowing random variation to accumulate. An important point here is that some of these features are used by the organism to adapt during its lifetime. There may be changes in the rate of cell division or the amount of a secreted molecule, or even the shape or mass of a bone, let’s say. But these are things that occur in the normal physiology of the organism, which is clearly under selection, so the system has evolved to accommodate them. Once you replace that physiological selection with more permanent genetic selection the mechanisms are already there to adjust to these changes.

## That’s beginning to sound like Lamarckism - in which Darwin, of course, also believed

Well, that’s right. The main questions about Darwinian evolution that people have focused on have been genetic questions; but the great doubt about Darwin in the period after *The Origin of Species*[1] was whether you would ever get a kind of genetic variation on which selection can act to produce a really complex adaptive structure: how likely is it that an eye would develop? It is easy to see that if an eye did develop, one could select for better and better eyes - but the origin of novelty is more challenging. So Darwinian evolution is clearly a good mechanism for improving things - but it is not necessarily a good mechanism for generating novelty. So the question for many people is how is novelty generated? And I think what modern biology has taught us is when you look at novelty under the microscope it’s not all that novel. The organism has the capacity to do a lot of different things physiologically, along with ways of regulating them with feedbacks and mechanisms that constrain them so that they’re non-lethal in the physiological realm, and it’s those kinds of mechanisms that help to explain the facility of generating novelty in evolution.

## Can you give an example of generation of novelty that actually isn’t so novel?

**Figure 1.**
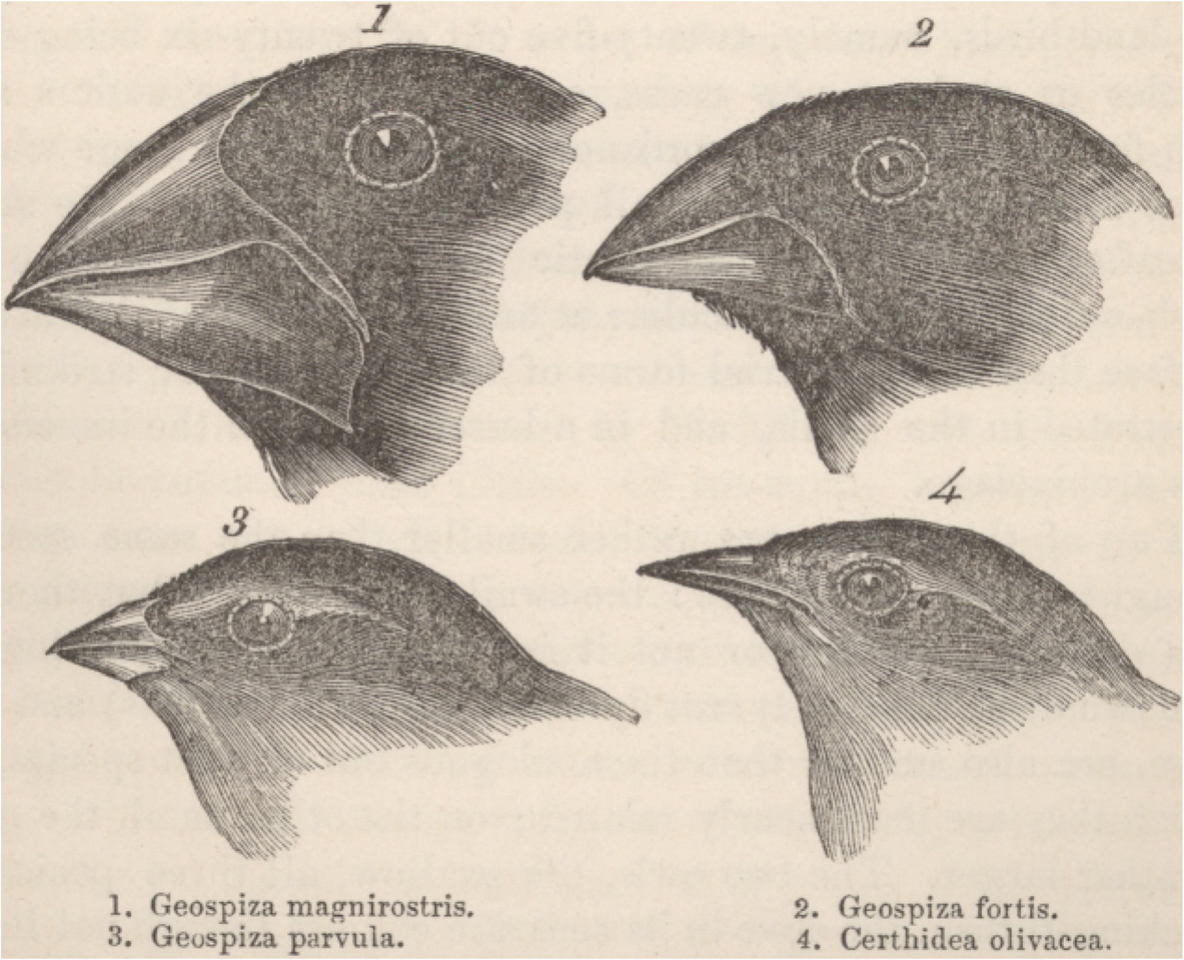
**Darwin’s or Galapagos finches.** Drawing from Darwin’s account of his observations on the voyage of HMS Beagle [2], showing the distinct adaptations of the beaks of the finches on Galapagos to different diets.

We can go to Darwin himself and his finches. You might imagine that to generate all the different beak shapes of the finches (Figure 1) [2] was actually difficult to do, and Darwin argued - and is one place where I think he may have overworked his theory a bit - that the changes had to be very, very small. For example if you had a change in the upper beak that was big and the lower beak was small, the beaks wouldn’t fit together; or if it was very large it wouldn’t fit into the head, or the muscles wouldn’t work, and then the whole system would be non-functional and the animal would die and wouldn’t make it into the next generation, even if it was headed in the right direction. So it does seem that to generate different beak shapes would be very difficult and need many, many small steps. Now, the problem with small steps is the smaller the steps the less their selective advantage. The other problem has been that in many cases the evolution took place in small populations that would not necessarily be able to accumulate all the mutations you need to generate the larger beaks, and certainly nothing very extreme. But now we know a lot about beaks - beaks are bony structures, they’re induced by neural crest cells that migrate into the head at various places, and one of the main things driving beak size is signaling through the BMP2/4 bone morphogenetic proteins. Cliff Tabin showed in experiments he did several years ago [3] that when you ectopically add BMP4 you get bigger beaks, and they work. The head changes its shape, the beaks change their shape. These kinds of pleiomorphic effects in evolution probably derive from the mechanisms for generation of the beaks during the growth of the baby birds. So you already have mechanisms in place for integrating information to make some sort of coherent change, and the evolution of novelty is not so difficult.

Even if you consider BMP itself, and how likely it is to change, you can see many ways that this could be done, because you know what you’re doing: you’re changing the signaling of BMP. You could be changing the amount of BMP or the number of BMP receptors. BMP itself is processed and secreted, and the processing or secretion can be changed; there are secreted BMP antagonists and they are subject to selection; all the elements in the pathway can be changed translationally, post-translationally, transciptionally; so all of the possible genetic modifications that could occur could be funneled into a process that has so many feedbacks in it that it ends up with the proper beak shape. In that view, what looks very unlikely in a small population of birds to generate beak shapes that go from pliers to forceps seems not so remarkable.

## Why do you think these things are important in a practical human context?

That’s always a good question. We’re very good at looking forward to things we want to understand, but we’re very poor at thinking about the historical roots to that understanding and realizing, for example, that fundamental discoveries have been the major mechanism by which we’ve been able to ask much better-defined and almost all important applied questions. So when we look at biology we think of it in terms of a very, very complex machine that we’re trying to describe and then to explain and ultimately to fix. And we can sort of explain how it might work, but we have trouble explaining why it’s as complex as it is. But very seldom do we look at the nature of that complexity. It’s not just complex, it’s not just something that was cobbled together randomly with no rhyme or reason to it. And the reason behind it almost certainly has to be the capacity of the system to evolve. So I think evolvability or the study of the properties of all biological systems that make it possible is the secret to understanding the systems themselves. And since a major goal of biology is to understand how the whole thing works, we might be better off understanding the principles on which it can evolve than just trying to describe its complexity without any reference at all to its past history.

## So you think this kind of thing is going to be important within our lifetimes in explaining, for example, the metabolic changes that take place in tumor cells where you’ve got a highly evolved system that is extremely complex, packed with feedback loops, and you don’t know what you’re doing to it most of the time?

People are right now thrilled to show how the complex adaptations of tumor cells are reflected in the cell’s genome. Of course some of that complexity is meaningful and some of it is not, and because genetic modification is so easy to study and is so easy to represent, we conveniently forget that it’s the phenotype that is really under selection, not the genotype. So I think even just trying to describe the genetic variation that underlies the development of a tumor independent of understanding the phenotypic consequences is really a mistake. But to understand the phenotypic consequences we have to understand how a cell is built and why it’s built one way and not another. That is a tall order. It is important to remember that a tumor cell is a cell that is taking advantage of the evolvable mechanisms that pre-existed in the organism to begin with. If we think in these terms it will help us understand how the seemingly random events that take place genetically end up with such clear-cut and coherent growth advantages and ways of avoiding the immune system and means to penetrate other tissues - without the genetic damage killing the tumor cells along the way, as might happen for a mechanical system if it were randomly modified.

## Is evolvability itself under selection?

This is the kind of question that if you’re not careful will get a lot of people angry with you because it seems to suggest the selection of a characteristic that is not immediately useful. But I think there are several ways to avoid that kind of heresy. One is to suggest that mechanisms of evolvability build on mechanisms that are used to generate physiological adaptability - that is, the processes that are used by the body to adapt to environmental conditions or growth or changes in nutrition. Once these processes are in place you make it easier for the organism to respond to genetic change, and to give meaningful outcomes instead of maladaptive ones. I don’t think anybody could argue about whether physiology is under selection, so by coupling the mechanisms involved in genetic change to existing physiological mechanisms you can make evolvability logically respectable. That’s one possibility for the evolution of evolvability. It is simply a byproduct of the evolution of physiological adaptability.

The other way to make sense of evolving evolvability is to point out that evolving robust adaptable mechanisms allows the accumulation of lots of genetic variation in a population. Evolvability is achieved by increasing the capacity to tolerate non-lethal phenotypic changes, which are exposed to selection, and not just non-lethal genotypic changes that aren’t. Non-lethal genotypic changes are well understood - the notion of neutral mutations is well established - but there are also non-lethal phenotypic changes: we’re all walking examples of non-lethal phenotypic variation. Now, because this variation is exposed to selection, mechanisms can evolve for suppressing the expression of variant genes that can lead to lethal phenotypes at some stages of development or in some circumstances, but without eliminating the variant genotype. Therefore, a byproduct of suppressing lethality in phenotypic variation is the inevitable accumulation of a lot more genetic variation in a population and that makes it much easier for that population to evolve.

## Can I just get you to clarify that - do you mean that because we’re phenotypically robust we can adjust to the expression of genetic variations that had been suppressed in a way that we couldn’t if we were hard-wired to produce an invariant phenotype?

**Figure 2.**
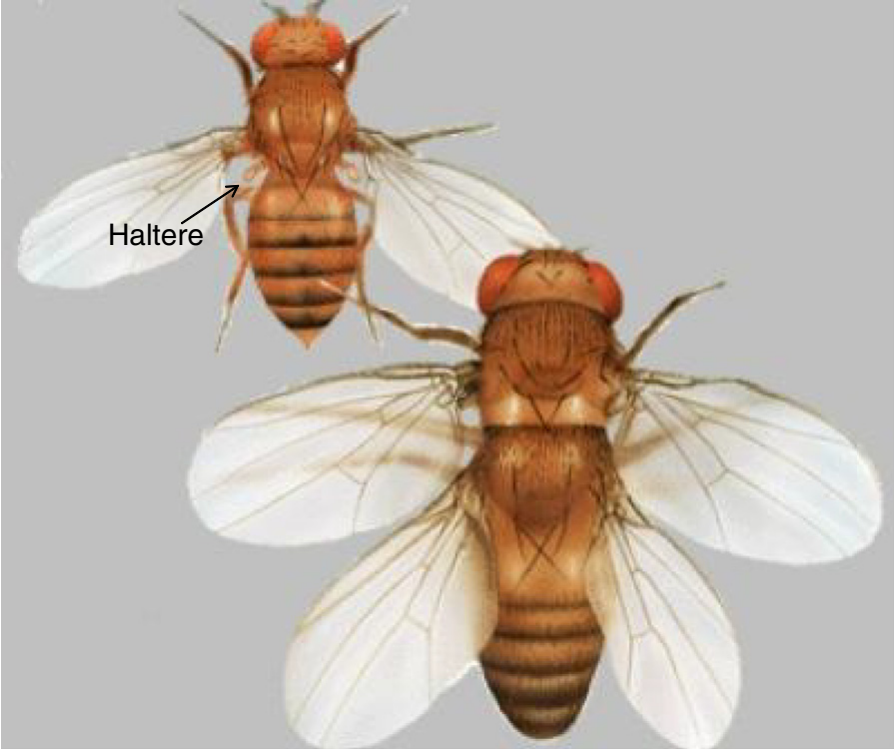
**The four-winged fruit fly.** In the homeotic mutant bithorax, the halteres, which are a balancing organ, are transformed into a second pair of wings. The same transformation can be achieved by treating fruit fly pupae with ether, and selection of fruit flies by this treatment over several generations for expression of the phenotype in the absence of ether led Waddington to his theory of genetic assimilation [4]. (Figure credit: FlyBase)

Right. And this gets us to mechanisms that Waddington was thinking about in the 1940s and 1950s [4], and Mary Jane West Eberhard has thought about more recently [5]. Waddington, in his classical work on genetic assimilation [4], started out with an out-bred population of *Drosophila* and selected for the four-winged phenotype, where the halteres are converted into an extra pair of wings (Figure 2). This change can be achieved genetically and is very well known as the homeotic transformation bithorax, but it can also be induced by treating the pupae with ether. Waddington kept selecting with ether, choosing intermediate phenotypes on the way to wings - so he was the selective force for generating the four-winged fly - and by constantly enriching for ether susceptibility he eventually got a whole population of flies that were four-winged in the absence of ether altogether. What we would say genetically now is that he was removing all the mechanisms or genes that were suppressing the four-winged phenotype, by first stressing the flies to reveal their underlying phenotypic plasticity and then selecting them. The extra wings have no obvious selective advantage in resisting ether, but this classic example illustrates the point that if you have processes that are already present but under suppression, then under stress you might see some of them emerge, and if you have fortuitous selection at the same time you can very quickly evolve. For example, if high temperature causes flies to have larger eyes, which is not itself an adaptation to temperature, and at the same time those flies are in dark caves, they could then very rapidly evolve larger eyes. So you can have the accumulation of neutral or even potentially maladaptive genetic variation in the population because you’re suppressing the phenotypic expression of variant forms, and that is one mechanism of evolvability.

Susan Lindquist has written about this more recently, in connection with prion-like proteins that provide a heritable resource of structural variability that can evolve new functions subject to cellular homeostatic restraints [6,7]. The molecular picture of genetic assimilation makes things much clearer and opens up very interesting applications in both evolution and human disease, but the fundamental notion of genetic assimilation was really invented by Waddington, and independently by Schmalhausen [8], in the 1940s.

Now the third is the most difficult example and that is the answer to the question why you would even evolve the capacity to change to conditions you’ve never encountered, and what does that even mean? There I would argue that, as we know from the geological record, we are the products of repeated extinctions and radiations. It’s not just a slowly growing network of lineages, whole branches were wiped out - in some cases most of the animals on the planet; and in other cases, locally organisms have been obliterated. So when you encounter a new niche, your ability to vary may be more important than your ability to compete. If you’re the first insect onto a desert island and you can evolve wings so that you can fly and teeth and claws so that you can eat the bark and burrow in the sand, if you will pardon the expression, the world is your oyster, so it’s possible that the successful lineages have built in a capacity to generate effective phenotypic variation more than the competition has, and we’re stuck with that history.

Ironically, when I’ve talked about that to people, of the three that’s the mechanism that traditional evolutionary biologists are afraid of because even though it seems plausible to me, it gets them closer to the heresies of inheriting acquired characteristics - although that’s not what it is at all.

Interestingly enough, if you talk to molecular biologists they like that mechanism the most. So there you have it: they’re all valid to some degree, I think, in terms of how evolvability could evolve under selection.

## You’ve thought a lot about evolvability. What have you done to investigate it?

When John Gerhardt and I wrote our first book, *Cells, Embryos and Evolution*[9], we felt a little guilty that we’d written a book on evolution and hadn’t actually worked on it, so we started working on an organism called a hemichordate, which was deep in the split between the early radiations of the deuterostome phyla (which we belong to). We were trying to understand what big changes allow whole new phyla to emerge. I think we’ve learned some things about what happened there, but it’s very hard to reconstruct evolutionary history from extant organisms.

**Figure 3.**
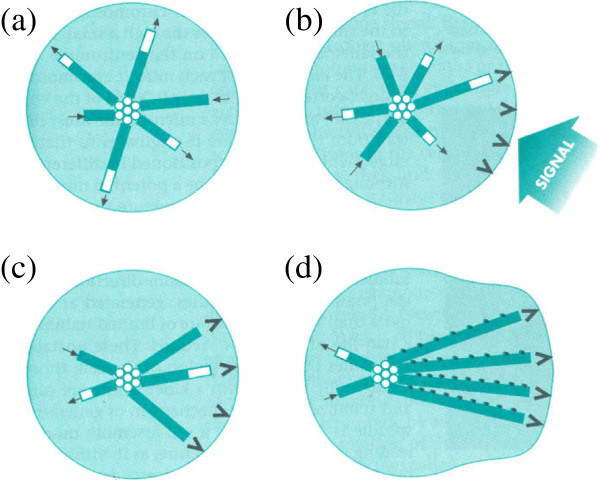
**Plasticity of microtubule arrays.** Microtubules are composed of tubulin subunits that can rapidly polymerize and depolymerize, and are thus said to show dynamic instability. They are here shown **(a)** growing rapidly from the cell center (centrosome), and shrinking more rapidly, producing a random organization. **(b)** A signal from the cell surface leads to **(c)** the selective stabilization of the growing and shrinking microtubule ends locally, and in **(d)** the microtubules are further stabilized by modifications that accumulate along their length. In this way, spatially random polymerization and local stabilization can lead to organized arrays of microtubules (here polarizing the cell), and allow the evolution of stable structures such as flagella and dynamic ones such as the mitotic spindle. Figure modified with permission from Figure 4-5 of Gerhart and Kirschner, *Cells, Embryos, and Evolution,* Oxford: Blackwell Science; © 1997.

The other direction I have taken has been to look at mechanisms, and the original inspiration for my work in this area was early work that Tim Mitchison and I did on dynamic instability of microtubules [10], because here was a process that generated all sorts of variation most of which was useless but all of which was available to future modification (Figure 3). That was a big insight for me. What understanding evolvability should be able to do is lead you to questions that have been overlooked but that are really fundamental because they relate more to evolvability than to the functional properties you’re trying to explain. So one of the things I’ve been very puzzled about recently is why recognition sequences are so small. For example, a eukaryotic transcription factor might recognize a DNA sequence six nucleotides long, and though there are hundreds of thousands of copies of that sequence in the genome only a few of them function in transcriptional regulation. The same is true for the amino acid sequences recognized by kinases for phosphorylation, and it also turns out to be true for ubiquitination, which is something that I’ve worked on. The point is that mechanisms that depend on such minimal information have tremendous potential for allowing the evolution of new functions or pathways. It’s so easy to make changes and create new sites; that’s one of the wonderful things about eukaryotic transcription.

## But then you need some additional mechanism to ensure specificity, don’t you?

Yes. It’s always been a puzzle why prokaryotes have transcription factor binding sites that are larger than those of eukaryotes even though eukaryotes have much more DNA and many more transcription factors. So we started looking at that as a puzzle, saying here is something that’s evolvable, but it also seems to carry as a consequence something that’s very negative for the organism - lack of specificity - and my colleague Ying Lu and I realized that organisms have in fact gone to great lengths to make transcription factor binding and phosphorylation and ubquitination specific and maintain their evolvability. There’s a trade-off between making something that is very robust and making something which is very changeable.

## What do you regard as being the major way in which this has been done by these systems?

Well I think to some degree John Hopfield [11] was going in the right direction with kinetic proofreading - some way in which energy could be put into the system to increase specificity, and he was right about how energy could be used to increase specificity to arbitrary levels; but he didn’t discuss the efficiency of the process. The more you increase specificity by these means the less efficient the system becomes. So the question is how to achieve both efficiency and specificity and as a byproduct have changeability. All the mechanisms anyone has thought of must use energy, so that you incrementally increase the specificity of the system in repeated energy-using steps rather than get all of it in one shot.

## You mean by adding components that bind to other components combinatorially, and making post-translational modifications that add new binding surfaces - or subtract them?

Right, with bells and whistles - adding extra components, modifications and intermediate steps. And that gets back to this question: why is biology as complicated as it is? If you go back to Jacob and Monod and look at transcriptional regulation again, the transcriptional regulation of lactose metabolism that they classically described [12] seemed to be a perfectly wonderful mechanism; but in eukaryotes metabolic regulation is very different and much less simple and elegant - and this isn’t because eukaryotes have a lot of time to waste doing unnecessary things, it’s because if eukaryotes are going to evolve they’re going to have to achieve the capacity to change very easily with small populations and also maintain their robust behavior. Bacteria have come up with great ways to do this in large populations with very little sex but eukaryotes have explored a different set of mechanisms operating with smaller populations under different initial and environmental conditions. So I think many of the processes that people work on in biology with features that are puzzling and seem inexplicable will turn out to have explanations in terms of evolvability.

## This is intellectually challenging and absorbing - but can you suggest how somebody entering biology might investigate evolvability in a way that would be productive for today’s career-driven biological scientists?

I’m making the assumption, which I hope is still true, that you can be working upstream of some final and perhaps undetermined application and still get support in our society for doing it. The impact of this sort of basic research is often ultimately greater than research close to application, but it’s hard for some people to see the connection to a translational outcome. I’m not worried about that. If we were interested in just translational outcomes we would have invested all our money into building better iron lungs instead of investigating the virus that caused polio in the first place.

There are two approaches that I think are interesting.

One is to look at development - embryology - in multiple and perhaps obscure organisms to try and understand how things might have changed, what was the underlying nature of the change. And not just what are the genetic differences. When you’re looking at very different organisms the genetic differences are huge, and if you’re looking at very closely related organisms, just varieties of different organisms, people would say you’re explaining something that’s uninterestingly trivial. We want to explain big things - how did our brain and hand develop, how did the heart develop, how did the placenta develop - all these things that make a big difference to us, and I think we can begin to do that. This takes experimental approaches. I doubt whether we can learn enough if we restrict ourselves to sequencing genomes. So I think that to explain these developments in terms of the properties of cell and developmental systems will unify biology into a set of common principles that can be applied to different systems rather than a number of special cases that have to be learned somehow by rote.

On the other side, I would say that when we look at problems that are baffling in their complexity, like transcription, like immunology, or even the cytoskeleton or protein secretion, not to mention the nervous system, all of these systems - if we think what are the properties of those systems, and study the mechanisms that are being employed and how they’re being modified to achieve their physiological function, we’ll have a better understanding of those systems, and maybe a better chance of modifying them and even understanding the pathology of the systems. So I think understanding the way in which evolvability can be reconciled with preserving present function is a way of knowing that will make it easier for people to understand biology.

## And do you think these principles can be extended beyond biology?

Yes, well, I’m kind of shy about going there. Darwin was not at all responsible for social Darwinism, to which his name was attached. It was a Victorian concept that got transformed into something much uglier later and tended to justify the winners and to denigrate the losers, and was used in those terms; and it’s still used in those terms. And so I’m always worried about using biology or the human body as a metaphor for other things. It’s a risky business. But I do think that some of the principles of evolvability that John Gerhart and I have discussed - particularly in our last book, *The Plausibility of Life*[13] - might have some applications beyond biology. It would be interesting to look at the behavior of systems - whether they’re economic systems or industrial systems or social systems or whatever kind of systems - in terms of biological principles, because these principles at least provide new metaphors for thinking about these things. I did once hear a talk from Carliss Baldwin at the Harvard Business School who argued that evolutionary principles could be very useful in thinking about how the organization of companies in very competitive technology sectors might determine their success or failure. For example, the exploratory behavior of certain systems, or weak linkage and modularity makes it easier to reconnect things and may contribute to success. It’s probably not for me to apply the principles of evolvability outside biology. But I am interested, because people I wouldn’t necessarily expect to be interested in evolvability have at least found it interesting to think about these principles. The three groups I hadn’t considered were computer scientists, architects, and people who study linguistics. The initial efforts of computer science to use biology and in particular genetic algorithms were very simplistic and didn’t take into account the capacity of certain systems to generate the proper kind of variation. And linguistics is another system where you have very powerful simple mechanisms that can adapt to novelty that had never previously been considered; so I would just say people might read about evolvability and see whether this inspires them to think about their own field differently.
